# Deletion of soluble epoxide hydrolase attenuates mice Hyperoxic acute lung injury

**DOI:** 10.1186/s12871-018-0490-z

**Published:** 2018-04-27

**Authors:** Li-Ping Liu, Bin Li, Tian-Kui Shuai, Lei Zhu, Yu-Min Li

**Affiliations:** 1 The Second Clinical Medical College of Lanzhou University & Key Laboratory of Digestive System Tumors of Gansu Province, Lanzhou, Gansu 730030 China; 2 Department of Critical Care Medicine, The First Hospital of Lanzhou University, Lanzhou, Gansu 730000 China; 3 The Donggang District of First Hospital of Lanzhou University, Lanzhou, Gansu 730030 China

**Keywords:** Soluble epoxide hydrolase, Keap1, Nrf2, Hyperoxia, Acute lung injury

## Abstract

**Background:**

Recent studies reported that soluble epoxide hydrolase (sEH) plays an important role in lung diseases. However, the role of sEH in hyperoxia-induced ALI is unclear.

**Methods:**

ALI was induced by exposure to 100% oxygen in an airtight cage for 72 h in wild-type (WT) and sEH gene deletion (EPHX2^−/−^) mice. ALI was assessed by the lung dry/wet ratio, alveolar capillary protein leak, and the infiltration of inflammatory cells in the lung.

**Results:**

Hyperoxia elevated sEH activity in WT mice. Simultaneously, epoxyeicosatrienoic acids (EETs) levels were decreased in WT mice exposed to hyperoxia. However, the level of EETs was increased in EPHX2^−/−^ mice exposed to hyperoxia. Hyperoxia induced pulmonary edema and inflammation were dampened in EPHX2^−/−^ mice compared with WT mice. Decreased expression of Kelch-like ECH-associated protein 1 (Keap1) was found in EPHX2^−/−^ mice exposed to hyperoxia. Hyperoxia-induced the expression of nuclear-factor erythroid 2-related factor 2 (Nrf2) was enhanced in EPHX2^−/−^ mice compared with WT mice. Simultaneously, the activities of heme oxygenase-1 and superoxide dismutase were elevated in EPHX2^−/−^ mice. The levels of reactive oxygen species were inhibited in EPHX2^−/−^ mice compared with WT mice exposed to hyperoxia.

**Conclusions:**

sEH is a harmful factor for hyperoxic ALI. The beneficial effect of sEH gene deletion is associated with the elevation of EETs and regulation of Nrf2/Keap1 signal pathway.

## Background

Acute respiratory distress syndrome (ARDS) is a common severe complication of sepsis with a complicated pathophysiological mechanism [1]. A mild form of ARDS was previously known as acute lung injury (ALI) [2]. Consistent with sepsis [3, 4], to date, there are no effective pharmacological interventions for ARDS [1, 5]. A recent animal study reported that inhibition of soluble epoxide hydrolase (sEH) reduces lipopolysaccharide-induced ALI [6]. sEH is a novel pharmacological target for inflammatory disorder [7]. For pulmonary diseases, inhibition or deletion of sEH reduced cigarette smoke-induced pulmonary inflammation [8], bleomycin-induced pulmonary fibrosis [9], angiotensin II-induced ALI [10]. These results suggest that sEH contributes to the pathogenesis of lung inflammatory disorders including ARDS.

Hyperoxia is a very high topic in medicine [11]. Recently, the WHO recommended the use of high fraction of inspired oxygen for the prevention of surgical site infections [12]. However, the hyperoxia may be a double-edged sword. Evidence has shown that hyperoxia causes ARDS [13]. The potential mechanism that is involved in the hyperoxia caused ARDS is complicated. Over generated reactive oxygen species (ROS) products cause an imbalance between oxidant and antioxidant. Antioxidant enzymes such as heme oxygenase (HO)-1 and superoxide dismutase (SOD) scavenge ROS playing an important role in antioxidant defense. The expression of antioxidant enzymes is regulated by nuclear-factor erythroid 2-related factor 2 (Nrf2). Nrf2 defends the lung from hyperoxic ALI [14].

As the sEH is suggested as a pharmacological target for ALI [6], we, therefore, investigate that whether the sEH plays any role in hyperoxic ALI in the present study, and the effect of sEH gene deletion on Nrf2 pathway.

## Methods

**Fig. 1:**
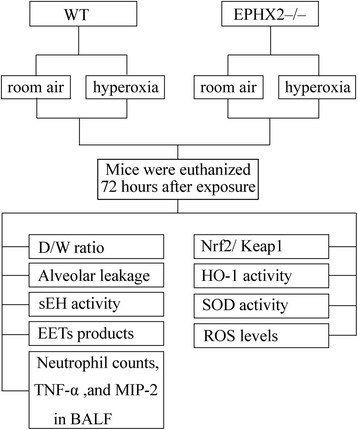
A diagram of the protocol. WT, wild-type mice; EPHX2−/−, soluble epoxide hydrolase (sEH) gene knockout mice; D/W ratio, lung dry/wet weight ratio; EETs, epoxyeicosatrienoic acids; TNF-α, tumor necrosis factor-α; MIP-2, macrophage inflammatory protein-2; BALF, bronchoalveolar lavage fluid; Nrf2, nuclear-factor erythroid 2-related factor 2; Keap1, Kelch-like ECH-associated protein 1; HO-1, heme oxygenase-1; SOD, superoxide dismutase; ROS, reactive oxygen species

The current study was performed in accordance with the Guide for the Care and Use of Laboratory Animals of Lanzhou University, and approved by the Animal Care and Use Committee of Lanzhou University (no. 20150520016). Eight- to ten- weeks old male C57BL/6 wild-type (WT) mice and sEH gene deletion (EPHX2^−/−^) mice were used in the present study. The C57BL/6 WT mice were obtained from Lanzhou University. EPHX2^−/−^ mice were back-crossed onto a C57BL/6 genetic background for more than ten generations as previously described [15]. The mice were housed in a temperature-controlled facility (22 ± 2 °C) and kept under a 12-h light/dark cycle. The animals were given free access to food and water. Hyperoxic ALI was induced by exposure of the mice to 100% oxygen in an airtight cage for 72 h, as described previously [16]. We performed the Evan’s blue (EB) dye technique 30 min prior to sacrifice to measure the pulmonary microvascular albumin-permeability as previously described [17]. The leak of EB dye-labeled albumin into the lung tissue was measured spectrophotometrically and calculated after sacrifice. Animals were euthanized at 72 h after hyperoxia exposure. The trachea was isolated by blunt dissection and a small-caliber tube was inserted into the airway and secured. Then, three volumes of 1 ml of phosphate buffered saline (pH 7.2) were instilled and gently aspirated. The fluid recovery rate was > 90%. The bronchoalveolar lavage fluid (BALF) was centrifuged at 1000×g for 10 min at 4 °C. The supernatant was analyzed for protein and cytokines. Cell counts were measured with a hemocytometer. Then, the lung was stored in liquid nitrogen at − 80 °C. Before use, the frozen lung tissue samples were crushed to powder with a mortar and pestle. A diagram of the protocol was shown in Fig. 1.

### Pulmonary water content measurement

Pulmonary tissues were weighed and, then, dried in a 70 °C oven for 48 h. Pulmonary water content was calculated as percent H_2_O = (1 – dry /wet) × 100%.

### Enzyme-linked immunosorbent assay (ELISA)

Tumor necrosis factor (TNF)-α and macrophage inflammatory protein (MIP)-2 were quantified using ELISA kits (R&D Systems) according to the manufacturer’s instructions.

The 11,12-epoxyeicosatrienoic acids (EETs) and 14,15-EETs are suggested as effective agents suppressing lung inflammation [18]. ELISA kits (Detroit R&D, Detroit, MI, USA) were used to determine concentrations of 11,12-EETs and 14,15-EETs and their stable metabolites 11,12-dihydroxyeicosatrienoic acids (DHETs) and 14,15-DHETs in lung tissues according to the manufacturer’s instructions.

The level of 8-Isoprostane was assessed by an 8-Isoprostane Oxidative Stress ELISA Kit (Detroit R&D, Michigan, USA) according to the manufacturer’s instructions.

### Measurement of SOD activity

The activity of SOD was measured by a SOD activity assay kit according to the manufacturer’s instructions (R&D Systems, Inc., MN, USA). Briefly, total 50 μg of protein samples were incubated with tetrazolium salt and xanthine oxidase enzyme for 20 min at 37 °C. Then, xanthine oxidase solution was added. The absorbance of the formazan salt was detected at 550 nm.

### Measurement of HO-1 activity

Lung tissues were harvested. The samples (50 μl of lung cytosol, 200 μl of lung supernatant, 20 μl of 1 mM heme solution, 200 μl of 2.75 mM NADPH solution, and 530 μl of 2 mM MgCl_2_ in 100 mM phosphate buffered saline (pH 7.4)) were incubated in a 37 °C water bath in the dark for 1 h. Then, the reaction was stopped by placement on ice. The absorbance of the sample was measured by spectrophotometer at 530 nm. The amount of bilirubin formed was calculated from the difference in absorbance at 530 nm. An NADPH-free reaction sample was served as an internal control [19].

### Western blot analysis

Homogenized lung tissues were lysed with lyses buffer containing protease inhibitors cocktail (Cell Signaling, Boston, MA, USA). BCA method was used to determine protein concentration (Pierce Chemical Co., TX, and USA). Total 50 μg protein samples were separated by 10% sodium codicil sulfate-PAGE, and were transferred to a hyoid-enhanced chemiluminescence’s (ECL) nitrocellulose membrane. The membrane was blocked in 5% skim milk powder at room temperature for one hour. Then, the membrane was blotted with the primary antibody (1:1000) against Nrf2 (Cambridge, MA, USA) and Kelch-like ECH-associated protein 1 (Keap1) (Santa Cruz Biotechnology Inc., Santa Cruz, CA,USA) overnight at 4 °C and the corresponding secondary antibody (1:5000) at room temperature for one hour. Finally the blots were visualized with an enhanced ECL western blot detection system (Habersham Pharmacia Biotech, Piscataway, NJ, USA). The band intensities were quantified using Image J 1.47v software (NIH.USA). Laming B and β-acting (Cell Signaling, Boston, MA, USA) were used as internal references for determination of nuclear and cryptozoic protein, respectively.

### Statistical analysis

Data are expressed as means ± SEM. Data are analyzed by using Graph Pad Prism 3.0 (Graph Pad Software). The two-tailed Student’s t-test for comparison between two groups. The one-way analysis of variance (ANOVA) followed by Bonferroni’s post hoc test for multiple comparisons. A *P* value of < 0.05 was considered to be statistically significant.

## Results

### Hyperoxia-triggered lung edema and alveolar leakage were attenuated in sEH knockout mice

**Fig. 2:**
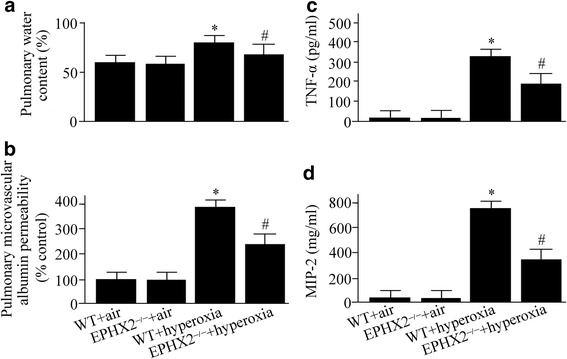
Wild-type (WT) and soluble epoxide hydrolase gene knock out (EPHX2^−/−^) mice exposed to room air or 100% oxygen for 72 h were euthanized. Lung edema (**a**), alveolar leakage (**b**), tumor necrosis factor (TNF)-α (**c**), and macrophage inflammatory protein (MIP)-2 levels (**d**) were measured. Data are represented as the mean ± SEM of three independent experiments. ^*^P < 0.05 vs. WT mice exposed to room air; ^#^ *P* < 0.05 vs. WT mice exposed to hyperoxia

The lung edema was measured by the dry to wet ratio of the lung. Our results showed that the dry to wet ratio was the same for both the groups exposed to room air (Fig. 2a). However, lung edema was triggered by hyperoxia exposure (Fig. 2a). As shown in Fig. 2a, the lung dry to wet ratio was inhibited in the sEH knockout mice compared with WT mice under hyperoxia.

The alveolar leakage was measured by the Evan’s blue dye technique. The alveolar albumin-permeability increased by 3.9-fold in the WT mice under hyperoxia compared with WT mice under air (Fig. 2b). The hyperoxia-induced alveolar leakage was markedly improved in sEH knockout mice (Fig. 2b).

### Hyperoxia-induced upregulation of TNF-α and MIP-2 was suppressed in sEH knockout mice

Seventy-two hours after hyperoxia exposure, the levels of TNF-α and MIP-2 were increased in the WT mice as evaluated by ELISA (Fig. 2c and d). However, the hyperoxia-induced upregulation of TNF-α and MIP-2 was inhibited by 43% and 66% in the sEH knockout mice compared with WT mice, respectively (Fig. 2c and d).

### Hyperoxia-induced infiltration of neutrophil was suppressed in sEH knockout mice

**Fig. 3:**
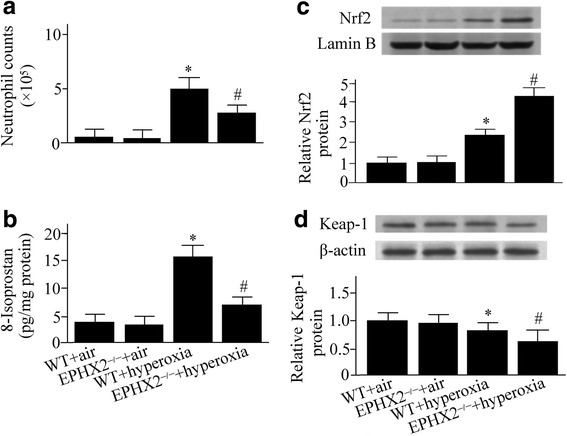
Wild-type (WT) and soluble epoxide hydrolase gene knock out (EPHX2^−/−^) mice exposed to room air or 100% oxygen for 72 h were euthanized. Neutrophil counts (**a**), 8-Isoprostane levels (**b**), nuclear-factor erythroid 2-related factor 2 (Nrf2) (**c**), and Kelch-like ECH-associated protein 1 (Keap1) (**d**) were measured. Lamin B and β-actin were used as internal references for determination of nuclear and cytosolic protein, respectively. Data are represented as the mean ± SEM of three independent experiments. ^*^P < 0.05 vs. WT mice exposed to room air; ^#^ *P* < 0.05 vs. WT mice exposed to hyperoxia

Neutrophil plays a vital role throughout the progression of ALI. We found that the neutrophil counts in BALF were increased in the WT mice under hyperoxia compared with the group exposed to room air (Fig. 3a). However, the hyperoxia-induced accumulation of neutrophil was reduced by 44% in the sEH knockout mice compared with WT mice (Fig. 3a).

### Hyperoxia-induced lipid peroxidation products were decreased in sEH knockout mice

The lipid peroxidation is an indicator for oxidative stress. Hyperoxia increased the incidence of 8-Isoprostan formation, an indicator for lipid peroxidation, in the lung in the WT mice (Fig. 3b). However, we found that the hyperoxia-induced elevation of 8-Isoprostane was inhibited in the sEH knockout mice compared with WT mice (Fig. 3b).

### Hyperoxia-induced activation of Nrf2/Keap1 pathway was enhanced in sEH knockout mice

As shown in Fig. 3c, elevated Nrf2 levels in the nucleus were detected by western blot analysis in the WT mice exposed to hyperoxia. The hyperoxia-induced upregulation of Nrf2 in the nucleus was enhanced in the sEH knockout mice compared with WT mice (Fig. 3c).

Keap1 is the inhibitor of Nrf2. Our western blot analysis showed a decrease in Keap1 in the cytoplasm in EPHX2^−/−^ mice compared with WT mice exposed to hyperoxia (Fig. 3d).

### Activities of antioxidant enzymes were increased in sEH knockout mice exposed to hyperoxia

**Fig. 4:**
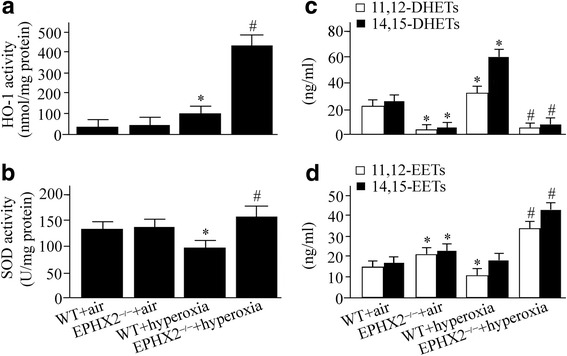
Wild-type (WT) and soluble epoxide hydrolase gene knock out (EPHX2^−/−^) mice exposed to room air or 100% oxygen for 72 h were euthanized. Heme oxygenase (HO)-1 activity (**a**), superoxide dismutase (SOD) activity (**b**), 11,12-dihydroxyeicosatrienoic acids (DHETs) and 14,15-DHETs levels (**c**), 11,12-epoxyeicosatrienoic acids (EETs) and 14,15-EETs levels (**d**) were measured. Data are represented as the mean ± SEM of three independent experiments. ^*^P < 0.05 vs. WT mice exposed to room air; ^#^ *P* < 0.05 vs. WT mice exposed to hyperoxia

In the WT mice, hyperoxia exposure increased the HO-1 activity by 2.8-fold, but reduced the SOD activity by 23%, respectively (Fig. 4a and b). However, both the HO-1 and SOD activities were increased in the sEH knockout mice compared with WT mice exposed to hyperoxia (Fig. 4a and b). There was no significant difference in the activities of HO-1 and SOD between the sEH knockout mice compared with WT mice exposed to room air (Fig. 4a and b).

### Hyperoxia-induced elevation of DHETs and reduction of EETs were inhibited in sEH knockout mice

sEH converts EETs to DHETs. The levels of 11,12-DHETs and 14,15-DHETs were used to determine the activity of sEH by an ELISA kit. As shown in Fig. 4c, hyperoxia exposure led to an increase in 11,12-DHETs and 14,15-DHETs products in WT mice. Seventy-two hours after hyperoxia exposure, the levels of 11,12-EETs were reduced; but, the 14,15-EETs levels were increased (Fig. 4d). However, there was no statistically significant in 14,15-EETs levels between WT mice exposed to hyperoxia and room air (Fig. 4d). Our ELISA analysis showed that both 11,12-EETs and 14,15-EETs levels were increased in the sEH knockout mice exposed to hyperoxia (Fig. 4d).

## Discussion

Hyperoxia is used to support critically ill patient [20]. However, hyperoxia causes elevation of ROS products, and activates inflammatory mediators [21]. Our data corroborate these findings. The ROS products were markedly increased in WT mice exposed to hyperoxia. It was simultaneously associated with an increase in pulmonary inflammation and edema. However, the hyperoxia-induced ROS products and lung injury were dampened in sEH gene knockout mice. Moreover, the activities of antioxidant enzymes and Nrf2 were enhanced in sEH knockout mice compared with WT mice. These results suggest that sEH is a harmful factor for hyperoxic ALI.

ROS cause toxicity to cellular components including DNA, lipids, and proteins, resulting cell swelling and cell membrane breakdown [22]. ROS generated in normal physiologic conditions did not cause harm, because the ROS can readily detoxified by antioxidants. The balance between oxidants and antioxidants is known as redox homeostasis. However, harmful stimuli such as prolonged hyperoxia exposure can disrupt this equilibrium. Excessive generated oxidants overwhelm the antioxidants leading to oxidative stress [22]. Our results are consistent with previous studies, hyperoxia exposure for 72 h induced ROS products as well as lung inflammation and edema. Antioxidant enzymes such as HO-1 and SOD play an essential role in maintenaning the balance between antioxidants and oxidants. Evidence has shown that SOD gene-modified mesenchymal stem cells attenuate acute radiation-induced ALI [23]. HO-1 is known as a vital antioxidant enzyme [24]. Upregulation of HO-1 protects the lung during ALI [25, 26]. In the present study, the activities of SOD and HO-1 were enhanced in sEH gene deleted mice. Simultaneously, the ROS products were reduced in EPHX2^−/−^ mice.

Nrf2 defends the lung from oxidative stress [14]. Nrf2 deficiency increases susceptibility to hyperoxic lung injury [27], and impairs the resolution of pulmonary inflammation [28, 29]. Likewise, deletion of Nrf2 in airway epithelium also exacerbates hyperoxic ALI and impairs the resolution of pulmonary inflammation [30]. These results suggest that Nrf2 is essential for protection against hyperoxic ALI. Nrf2 is a transcription factor which activates many anti-oxidant genes via phosphorylation and dissociation from the cytoplasmic inhibitor, Keap1. In the present study, the Keap1 was inhibited in sEH knockout mice compared with WT mice exposed to hyperoxia. It was simultaneously associated with an increase in the nuclear level of Nrf2.

Inhibition or deletion of sEH is associated with dampened oxidants products [31, 32]. However, the underlying mechanism has not been fully characterized. sEH saves EETs which has antioxidant role [33]. EETs inhibit Bach-1, a negative regulator of HO-1 expression [34], which in turn activates HO-1 expression. Our data are consistent with previous studies, the EETs levels and HO-1 activity were increased in sEH knockout mice compared with WT mice exposed to hyperoxia. Moreover, our results suggest that the benefit of sEH gene deletion in hyperoxic ALI is associated with activating Nrf2/ Keap1 pathway.

## Conclusions

sEH is a harmful factor for hyperoxic ALI. The beneficial effect of sEH gene deletion is associated with the elevation of EETs and regulation of Nrf2/Keap1 signal pathway.

## Abbreviations

ALI: acute lung injury
ANOVA: analysis of variance
ARDS: acute respiratory distress syndrome
BALF: bronchoalveolar lavage fluid
D/W ratio: lung dry/wet weight ratio
EETs: epoxyeicosatrienoic acids
ELISA: enzyme-linked immunosorbent assay
HO-1: heme oxygenase-1
Keap1: Kelch-like ECH-associated protein 1
MIP-2: macrophage inflammatory protein-2
Nrf2: nuclear-factor erythroid 2-related factor 2
ROS: reactive oxygen species
sEH: soluble epoxide hydrolase
SOD: superoxide dismutase
TNF-α: tumor necrosis factor-α
